# Prevalence, Knowledge, Attitudes, and Perceptions About E-Cigarette Smoking Among Students in the Dental Fields in Jordan

**DOI:** 10.1155/ijod/6521183

**Published:** 2025-03-25

**Authors:** Suhair R. Obeidat, Zain A. Malkawi, Omar F. Khabour, Amani Gh. AlSa'di

**Affiliations:** ^1^Department of Applied Dental Sciences, Faculty of Applied Medical Sciences, Jordan University of Science and Technology, P.O. Box 3030, Irbid 22110, Jordan; ^2^Department of Medical Laboratory Sciences, Faculty of Applied Medical Sciences, Jordan University of Science and Technology, P.O. Box 3030, Irbid 22110, Jordan; ^3^Jordan Food and Drug Administration, Irbid, Jordan

## Abstract

**Introduction:** Tobacco use is common in Jordan, with electronic cigarettes (e-cigarettes) becoming more prevalent, especially among the youth. Despite this trend, very few studies have been conducted on e-cigarette use among dental students, who should be more informed about the consequences of e-cigarette use on oral health. This study aimed at assessing the prevalence, knowledge, attitude, and perception of e-cigarette smoking among Jordanian dental students with respect to its effects on oral health.

**Methods:** The study utilized a cross-sectional survey method and used a convenient sampling approach. The study included 645 students from Dentistry Schools in Jordan.

**Results:** The most popular smoking type among participants was waterpipe. A total of 16% of students reported smoking e-cigarettes. The majority of e-cigarette users reported initiation of smoking at 17–18 years of age. Most users thought that e-cigarettes are less harmful compared to regular cigarettes, while 87% of them acknowledged the addictive nature of the product. Students demonstrated good awareness of the deleterious effects of e-cigarettes on oral health, and two-thirds of them expressed concern about its effects on general health.

**Conclusions:** The prevalence of e-cigarette smoking among dental students in Jordan is high and necessitates health education programs to increase awareness of the associated health risks among young adults, specifically in educational settings.

## 1. Introduction

Smoking is one of the biggest public health problems affecting the world today and a preventable cause of premature death. Electronic cigarettes (e-cigarettes) are a new class of nicotine products that began to emerge between 2006 and 2009 [1]. Smoking is rapidly increasing in developing countries, including Jordan [2–4]. For this reason, Jordan adopted the “National Tobacco Control Strategy for 2017–2019,” which relies on the World Health Organization's tobacco control measures and aims to reduce smoking in Jordan by about a third in 2025 [5].

E-cigarette use is marketed as a healthier/less toxic way to tobacco products [6]. An e-cigarette (vaping) is an electric device that heats a liquid mixture containing nicotine to produce an aerosol, which is then inhaled by users in a way that mimics traditional tobacco smoking. There are many types of electronic smoking, such as vape pens and vaporizers, which are called “electronic nicotine delivery systems (ENDSs)” [7]. Many conventional tobacco users have turned to e-cigarettes to quit smoking. In addition, e-cigarettes are increasingly popular among adolescents and young adults [8].

The liquid used in e-cigarettes contains two humectants: propylene glycol and glycerin [9]. Oxidation and decomposition of the propylene glycol and glycerin in e-liquid by heating leads to the formation of several harmful compounds, including formaldehyde, acetaldehyde, and acrolein [10]. These compounds have toxic effects, as reported by several studies [11–13]. For example, formaldehyde and acetaldehyde are classified as probable carcinogenic to humans. While acrolein is a powerful cardiovascular agent and also causes lung injury and nasal allergies [13, 14].

A report in 2019 by Beauval et al. [9] found that e-cigarette use leads to a higher level of nicotine dependence than traditional tobacco smoking. Furthermore, studies reported that e-cigarettes cause more addiction among adolescents and young adults than traditional cigarettes [14–16]. The addictive nature of e-cigarettes is related to nicotine content and preference factors (such as flavors). However, the majority of e-cigarette users believe that their health would be better if they vape instead of tobacco smoking [17].

To better characterize factors associated with e-cigarette use, studies should focus on populations in which e-cigarette use is common. Therefore, the aim of the current study is to examine the prevalence, knowledge and attitude of e-cigarette users among students of dentistry, dental hygiene, and dental technology in Jordan.

## 2. Methodology

A cross-sectional survey approach was used to investigate the knowledge, beliefs, and attitudes of Jordanian dental, dental hygiene, and dental technology students toward e-cigarette smoking. Institutional Review Board approval was obtained at the Jordan University of Science and Technology (JUST) before conducting the study (IRB reference: 15/153/2022). Data were collected anonymously, and thus, the privacy and confidentiality of the participants were maintained. Participation in the study was voluntary and informed consent was taken in accordance with JUST regulations.

A questionnaire was distributed using a convenient sampling technique to all registered Jordanian students (levels 2–5) from dental-related academic programs, including the Departments of Dentistry, Dental Hygiene, and Dental Technology at JUST. Those in the first year are not included because they are taking core university courses. Students were recruited from classrooms, teaching laboratories, practical clinics, and student breakouts.

The questionnaire was divided into sections, including participant demographics (age, gender, school year, academic achievement, personal income, and marital status), e-cigarette smoking status (types, magnitude of use, history of use), and perceptions, knowledge, and attitudes toward e-cigarette smoking. The questionnaire was revised by expert colleagues to check its clarity, and relevance. In addition, the validity and reliability of the questionnaire were tested by applying it to a sample of 20 students. Reliability was tested and confirmed using a Cronbach's *α* > 0.80. An online cover page was used to explain the purpose and the importance of the study to participants and to provide instructions for completing the questionnaire correctly. The questionnaire included ~50 questions in a multiple-choice format. Participants took 12–20 min to complete and return the questionnaire.

### 2.1. Data Handling and Statistical Analysis

Data were analyzed using Statistical Package for the Social Sciences (Chicago, IL, USA). Frequencies and percentages were generated. In addition, the Pearson Chi-square test was used appropriately to compare subgroups. Multivariable binary logistic regression was performed to examine predictors associated with e-cigarette smoking use. Odds ratios (ORs) and 95% confidence intervals (CIs) were estimated from the models. The significance level was set at (*p* < 0.05).

## 3. Results

A total of 645 students filled out the questionnaire. The majority of students were female (68.8%), from the Department of Dentistry (76.7%), and had a family income of >1000 Jordan dinars per month (Table 1). Nearly two-thirds of the sample came from northern Jordan (Table 1).

### 3.1. Oral Hygiene Status of the Study Sample

About 8.0% of the sample reported experiencing halitosis, with a significantly higher incidence among the e-cigarette smokers compared to the nonsmokers (*p* < 0.01). Similar findings were found for dental caries and tooth loss due to caries/periodontal disease (*p* < 0.01, Table 2).

### 3.2. Smoking History Among Study Sample

Approximately 37.0% of students had tried waterpipe smoking, followed by e-cigarettes (24.2%) and traditional cigarettes (21.4%). The prevalence of current smoking among the sample was 33.0% (Table 3). The most common type of current smoking among students was waterpipe (24.0%), followed by e-cigarettes (16.0%) and traditional cigarettes (9.6%) (Table 3).

### 3.3. E-Cigarettes Smoking Status Among Study Population

**Figure 1:**
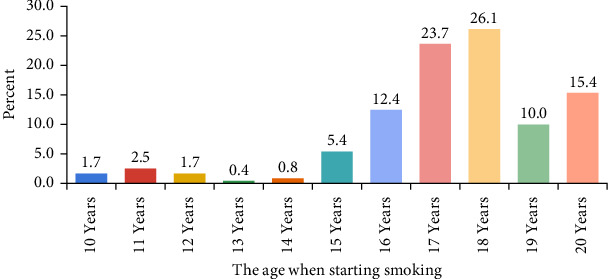
The age of initiation of smoking habit (*N* = 241). The majority of student smokers admitted that they started smoking between the ages of 17 and 18.

The majority of smokers reported smoking initiation using waterpipes (27%) (Table 3). About 50% of student smokers admitted that they started smoking between the ages of 17 and 18 (Figure 1). A smaller percentage of students reported smoking at an earlier age, ranging from 10 to 16 years. Specifically, initiation begins at a lower rate around 10–11 years of age (1.65% and 2.5%, respectively), gradually increasing through early adolescence and reaching 12.44% by age 16 (Figure 1). The data also shows a decline in smoking initiation after the age of 18 years. This suggests that most students who choose to smoke have already done so in late adolescence or early adulthood.

The results showed that 16% of students reported their current use of e-cigarette smoking, with the majority (70%) starting this type of smoking for a period ranging from 1 to 3 years and after giving it a try (67.0%, Table 4). In addition, about 50% of users believe that e-cigarettes are less harmful than traditional smoking. Moreover, most users reported that their source of knowledge about e-cigarettes was from their friends and social media (Table 4).

The Pennsylvania E-Cigarette Dependence Index indicates that 46% of e-cigarette users show a moderate level of dependence. Nearly half (52%) of e-cigarette smokers reported an intention to quit smoking, with most of them reported that the health effect of e-cigarettes was the primary reason for their desire to quit smoking. Other reasons cited by the study sample for quitting smoking were that e-cigarettes are addictive and that they are expensive (Table 4).

As for nonsmokers, about half of them attributed the reason for not smoking to religious reasons, while the majority of students (74.3%) said that they do not use e-cigarettes because of their health effects.

### 3.4. E-Cigarettes Beliefs and Perceptions and Knowledge

The majority of the study sample stated that they believe that e-cigarettes are more likely to cause addiction (87.4%) and are a public health concern (78%). In addition, the majority reported that e-cigarettes smoking should be prohibited at work and public places (Table 5). Moreover, the results revealed that about two-thirds (67.1%) had good knowledge regarding e-cigarette smoking and its negative impact on oral health (Table 5).

Multivariate analysis of factors associated with e-cigarette smoking was investigated and found that gender, student beliefs, and total knowledge were significant factors affecting the e-cigarettes smoking habit (Table 6). Specifically, male students were seven times more likely than female students to be e-cigarette smokers (OR = 7.248, 95% CI = 3.99–13.17, *p* < 0.01). Compared with students who believed that e-cigarette use was a public health concern, those who did not believe this assumption were 2.7 times more likely to smoke e-cigarettes (OR = 2.738, 95% CI = 1.468–5.108, *p* < 0.01). Students who reported that they did not believe in the regulation of e-cigarettes in public places were 2.2 times more likely to smoke e-cigarettes than those who did (OR = 2.234, 95% CI = 1.255–3.975, *p* < 0.01, Table 6). Students with poor knowledge (<50) about the negative impact of e-cigarettes on oral health were twice as likely to smoke compared to those with better knowledge (OR = 2.16, 95% CI = 1.042–4.478, *p*=0.038, Table 6).

## 4. Discussion

There is a lack of conducted studies about the prevalence and risk indicators of e-cigarettes among Jordanian universities' students. The limitations and weakness of the current study were that it was conducted during a short time due to the lack of time by using the convenient sample technique instead of using simple random sampling technique or other types of randomized base methods, which may affect the generalization of the results to the target group. Not only that, but also, the studies that use the self-structured questionnaire may have subjective answers that differ from one society to another, and if we compare the current results with results in other societies, we will find it difficult to make such comparisons due to the difference in the composition of the society, the mentality of the society, the method of collecting samples, the method of statistical analysis and other factors.

The purpose of this study was to evaluate the use, perceptions, knowledge, and attitude of e-cigarette smoking among Jordanian dental students. The study found that 16% of the study sample admitted to smoking e-cigarettes, the majority of whom were males, and the most common type of smoking among students was waterpipe. A multinational survey conducted among dental students from various countries found that the prevalence of e-cigarette use was high and was related to country and gender [18]. For example, studies conducted among dental students in Saudi Arabia found that 26%–43% of participants had ever used e-cigarettes, with higher percentages among males [19, 20]. A study conducted among university students aged 18–24 years in New Zealand found that daily e-cigarette use was significantly higher in males than among females [21]. The gender difference in smoking habits is often attributed to different social, cultural, and marketing factors. In Jordan and most Arab countries, smoking is considered less acceptable for females than for males.

Notably, e-cigarette smokers in our study reported higher rates of oral health problems, including halitosis, tooth decay, and tooth loss, underscoring the potential harmful effects of e-cigarette use on dental health. This result is consistent with previous studies that indicated the negative health effects of e-cigarettes on oral health. For example, Alhajj et al. [18] noted that e-cigarette users reported a significantly higher prevalence of dry mouth and black tongue, which was attributed to the high viscosity of the e-liquid promoting colonization of *Streptococcus mutans*, a major causative agent of dental caries. According to a fact sheet published by the International Dental Federation (FDI), there is not enough data to establish a direct link between poor oral health conditions and e-cigarette use. However, recent systematic evaluations have shown that the most common effects on oral health are gum disease and mouth and throat irritation [22]. Epidemiological studies highlight concerns about dry mouth, irritation, and gum disease. In addition, microbiological studies have suggested that e-cigarette users have a distinct microbiome, and there is some evidence that this may be more pathogenic compared to nonusers [23].

Regarding students' knowledge of e-cigarettes, a noteworthy finding is that more than 50% of e-cigarette smokers believe that e-cigarettes are less harmful than traditional smoking, indicating the influence of public perception and advertising on their choices. In a similar way, a study conducted among university students in Jordan revealed various motivations for their early adoption of e-cigarettes. Reasons included efforts to stop smoking traditional cigarettes, fascination with e-cigarettes, and the belief that e-cigarettes represent a less risky alternative to traditional smoking [7]. In another study, students in the colleges of public health, pharmacy, and nursing at the University of Arkansas in the United States showed that there is a significant correlation between the use of e-cigarettes to quit smoking, the belief that they are less harmful than traditional tobacco, and the preference for less regulation over e-cigarettes, with actual consumption of e-cigarettes [24]. E-cigarette manufacturers and marketers often claim that their products are less harmful than traditional cigarettes. These claims can lead students to believe that e-cigarettes are a healthy option. Peer pressure and social influence play a significant role. If students see their friends or peers using e-cigarettes and perceive it as a socially acceptable or safer choice, they may be more inclined to try it.

In addition, our results also showed that more than 50% of e-cigarette users expressed their intention to quit smoking, primarily due to the harmful effects of e-cigarettes on health and the addictive nature of these products. It is essential that individuals acknowledge the potential health risks associated with e-cigarettes. The percentage of US adults who believe e-cigarettes are as dangerous as or more harmful than traditional cigarettes rose significantly between 2012 and 2017 [25].

Regarding students' attitudes, beliefs, and perceptions, the majority of students believed that e-cigarettes were addictive and considered e-cigarette use to be a public health concern. The majority supported regulating e-cigarettes in workplaces and public places. In addition, students who had better knowledge about the negative effects of e-cigarettes on oral health were less likely to smoke, underscoring the role of awareness and education in shaping behavior. In the same context, a study conducted among college students in the United States revealed that the perception of harm associated with e-cigarettes was significantly lower among individuals who used cigarettes exclusively or both cigarettes and other substances compared to those who did not use anything [26]. It appears that individuals who use e-cigarettes may not be fully aware of the potential risks that come with their use. Health organizations and professionals often emphasize the addictive properties of nicotine found in e-cigarettes. Warnings about addiction and its impact on health can reinforce these beliefs among students. Schools and colleges offering educational programs about the dangers of e-cigarettes can influence students' understanding. Accurate, evidence-based information can dispel myths and contribute to the perception of e-cigarettes as addictive and harmful.

The findings of the present study emphasize the immediate need for having stronger public health policies and educational programs on e-cigarette use among students and the general population at large, and there is an apparent need for training on the health risks of e-cigarettes so that future dental professionals can properly advice patients.

The cross-sectional nature, the convenient sampling approach, reliance on self-reporting data, and the study being carried out at a single institution imply caution in generalizing the results. In the future, longitudinal studies combined with diverse populations and clinical health assessments should be integrated to determine better long-term health outcomes from e-cigarette use. Furthermore, qualitative work could be undertaken to understand why e-cigarettes are so popular. Intervention research then needs to address the best methods to reduce consumption and encourage smoking cessation. These are critical steps necessary to achieve evidence-based public health strategies to reduce e-cigarette prevalence among young adults.

## 5. Conclusion

The results of the current study highlight some of the factors that influence e-cigarette use among dental students in Jordan. It is recommended that educational programs should be developed to increase awareness among students about the potential health risks associated with the use of e-cigarettes, including nicotine dependence. In addition, regulations should be implemented to control the use of e-cigarettes in public places and workplaces. The results can be used in interventions that target e-cigarette use among university students.

## Figures and Tables

**Table 1:** Distribution of sociodemographic variables of the study sample (*N* = 645).

Variables	*N* (%)
Sex Female Male	444 (68.8) 201 (31.2)
Academic program Dentistry Dental auxiliary (DH + DT)	495 (76.7) 150 (23.3)
University level Second year or less Third year Fourth year Fifth year or more	180 (27.9) 176 (27.3) 205 (31.8) 84 (13.0)
Family monthly income ≤500 JD/month 500–1000 JD/month >1000 JD/month	82 (12.7) 228 (35.3) 335 (51.9)
Residency Northern Jordan Middle + southern Jordan	423 (65.6) 222 (34.4)

**Table 2:** Oral hygiene status of study sample by e-cigarettes smoking (*N* = 645).

Oral hygiene status variable	*N* (%)	E-cigarettes smoking status		*p*-Value Person χ^2^ test
		No (*N*%)	Yes (*N*%)	
Halitosis No Yes	594 (92.1) 51 (7.9)	511 (94.3) 31 (5.7)	83 (80.6) 20 (19.4)	Value = 22.3; *p* < 0.001
Dental caries No Yes	282 (43.7) 363 (56.3)	249 (45.9) 293 (54.1)	33 (32.0) 70 (68.0)	Value = 6.8; *p* < 0.001
Tooth loss due to caries or periodontal diseases No Yes	514 (79.7) 131 (20.3)	447 (82.5) 95 (17.5)	67 (65.0) 36 (35.0)	Value = 16.24; *p* < 0.001

**Table 3:** Distribution of variables related to smoking history (*N* = 645).

Variable	*N* (%)
Have you ever used any of the following smoking types? Cigarettes smoking Waterpipe smoking E-cigarettes	138 (21.4) 240 (37.2) 156 (24.2)
What is your status regarding smoking (any type)? No Yes E-cigarettes Cigarettes Waterpipe	432 (67.0) 213 (33.0) 103 (16.0) 62 (9.6) 155 (24.0)
What is the first type of smoke that you used when you started smoking? None of them Cigarette smoking Waterpipe E-cigarettes smoking	369 (57.2) 75 (11.6) 175 (27.1) 26 (4.0)

**Table 4:** Distribution of variables related to e-cigarettes smoking habits (*N* = 645).

Variable	*N* (%)
Are you e-cigarette smoker No Yes	542 (84.0) 103 (16.0)
Duration of smoking e-cigarettes <1 year 1–3 years ≥4 years	12 (11.7) 72 (69.9) 19 (18.4)
The main reason for smoking e-cigarettes at the first time Avoid tobacco smoking prohibition in public places I had already quit conventional smoking; I wanted to avoid set back Just to give it a try (curiosity) Reduce conventional smoking exposure E-cigarettes are less expensive than tobacco cigarettes E-cigarettes are less harmful than conventional smoking On the recommendation of friends	32 (31.1) 31 (30.1) 69 (67.0) 37 (35.9) 28 (27.2) 53 (51.5) 62 (60.2)
From where do you know about e-cigarettes Radio/TV/newspaper Family members Relatives Friends Social media Internet search	17 (16.5) 58 (56.3) 52 (50.5) 88 (85.4) 60 (58.3) 33 (32.0)
Dependence index scores categories Low dependence Medium dependence High dependence	28 (27.2) 47 (45.6) 28 (27.2)
Desire to quit e-cigarettes No Yes	50 (48.5) 53 (51.5)
Reason to quit e-cigarettes E-cigarettes are harmful to health E-cigarettes cause nicotine dependence To save money E-cigarettes are socially not accepted	44 (83.0) 30 (56.6) 29 (54.7) 21 (39.6)
Reasons for not using e-cigarettes E-cigarettes are harmful to health E-cigarettes are expensive Because I have asthma E-cigarettes are not accepted socially Nobody in the family uses e-cigarettes Nobody has encouraged me to use e-cigarettes I tried e-cigarettes but did not like it I do not use e-cigarettes for religious reasons	402 (74.3) 107 (19.8) 38 (7.0) 102 (18.9) 91 (16.8) 179 (33.1) 59 (10.9) 298 (55.1)

**Table 5:** E-cigarettes beliefs and perceptions and knowledge score (*N* = 645).

E-cigarettes beliefs and perceptions and knowledge questions	*N* (%)
In your opinion, e-cigarettes is Not addictive Addictive	81 (12.6) 564 (87.4)
E-cigarettes use is a public health concern No Yes	141 (21.9) 504 (78.1)
E-cigarettes should be regulated in work and public places No Yes	175 (27.1) 470 (72.9)
E-cigarettes knowledge score Poor knowledge (≤50) Intermediate knowledge (51–70) Excellent knowledge (>70)	95 (14.7) 117 (18.1) 433 (67.1)

**Table 6:** Multivariate binary logistic regression model of predictors associated with e-cigarettes smoking habit among the study sample.

Predictors	Odds ratio (95% CI)	*p*-Value
Gender (male versus female)	7.248 (3.99–13.17)	<0.001
Academic program (dental auxiliary versus dentistry)	1.187 (0.636–2.218)	0.590
Having halitosis	1.749 (0.775–3.946)	0.178
Having dental caries	1.435 (0.798–2.579)	0.227
History of lost teeth (yes versus no)	1.553 (0.834–2.893)	0.165
Believing that e-cigarettes use is a public health concern (no versus yes)	2.738 (1.468–5.108)	<0.01
Believing that e-cigarettes should be regulated in work and public places (no versus yes)	2.234 (1.255–3.975)	<0.01
Knowing/reading components of liquid (yes versus no)	1.716 (0.824–3.574)	0.149
Total oral health effects of e-cigarettes' knowledge (≤50 versus >50)	2.16 (1.042–4.478)	<0.05

## Data Availability

Raw data can be made available upon request from the corresponding author.
